# Efficacy and Safety of Weekly Calcifediol Formulations (75 and 100 µg) in Subjects with Vitamin D Deficiency: A Phase II/III Randomised Trial

**DOI:** 10.3390/nu16223796

**Published:** 2024-11-05

**Authors:** Esteban Jódar-Gimeno, Jose Luis Pérez-Castrillón, Ján Nociar, Michal Lojka, Dimitar Nikolov, Fernando Cereto-Castro, Snežana Novković, Umberto Tarantino, Nadia Mehsen-Cetre, Paula Arranz, Cristina Martínez Ostalé, Aintzane García-Bea, Inmaculada Gilaberte

**Affiliations:** 1 Department of Endocrinology and Nutrition, Quirónsalud Madrid University Hospital, 28223 Madrid, Spain; esteban.jodar@gmail.com; 2 Department of Internal Medicine, Río Hortega University Hospital, 47012 Valladolid, Spain; uvacastrv@gmail.com; 3 Department of Cardiology, General Hospital with Polyclinic Lučenec n.o., 984 01 Lučenec, Slovakia; nociar@avexis.sk; 4 Ordinace MediFem, s.r.o., 415 01 Teplice, Czech Republic; lojkam@gmail.com; 5 Medical Center-1-Sevlievo EOOD, 5400 Sevlievo, Bulgaria; dr.dimitar.nikolov@gmail.com; 6 Department of Internal Medicine, Hospital Quirón Barcelona, 08023 Barcelona, Spain; fercereto@gmail.com; 7 Department of Internal Medicine, Institute of Rheumatology, 11000 Belgrade, Serbia; snekino@yahoo.com; 8 Department of Orthopaedics and Traumatology, Policlinico Tor Vergata Foundation, 00133 Rome, Italy; umberto.tarantino@uniroma2.it; 9 Rheumatology Department, CHU Pellegrin, 33076 Bordeaux, France; nadia.mehsen@chu-bordeaux.fr; 10 Clinical Research Department, FAES FARMA, 48940 Leioa, Spain; parranz@faes.es (P.A.); cmartinez_b@faes.es (C.M.O.); 11 Medical Affairs Department, FAES FARMA, 48940 Leioa, Spain; agarciabea@faes.es

**Keywords:** vitamin D deficiency, calcifediol, weekly, phase II/III, clinical trial, efficacy, safety

## Abstract

Background/Objective: Optimal vitamin D levels are required for bone health and proper functionality of the nervous, musculoskeletal and immune systems. The objective of this study was to assess the efficacy and safety profiles of new weekly calcifediol formulations with the potential to improve adherence and outcome. Methods: A Phase II-III, double-blind, randomized, multicentre trial (EudraCT 2020-001099-14 and NCT04735926). Subjects were randomized 2:2:1 to calcifediol 75 µg, 100 µg and placebo. 25(OH)D levels were measured at 4, 16, 24, 32 and 52 weeks. The main outcome was the percentage of subjects who achieved a response defined as 25(OH)D levels ≥20 ng/mL and/or ≥30 ng/mL at week 16. Results: 398 subjects (51.1 ± 15.96 years, 74.2% females, 98.7% Caucasian) with plasma 25(OH)D levels between 10 and 20 ng/mL were randomized. A total of 376 subjects completed 16 weeks of treatment, and 355 subjects completed the study. Six patients withdrew due to an adverse event, all unrelated to treatment. At week 16, 93.6% and 74.4% of subjects receiving calcifediol 75 µg achieved response levels of ≥20 ng/mL and ≥30 ng/mL, respectively. The calcifediol 100 µg group showed 98.7% and 89.9% of responders for ≥20 ng/mL and ≥30 ng/mL, respectively. Both calcifediol groups showed superiority over placebo at each response level at all time points analyzed (*p* < 0.0001). Calcifediol treatments increased 25(OH)D levels from baseline to week 24 and remained stable thereafter. The frequency of treatment-emergent adverse events was balanced between groups. Conclusions: New weekly calcifediol 75 and 100 µg formulations showed an effective and sustained response with a good long-term safety profile.

## 1. Introduction

Vitamin D deficiency is a worldwide concern. A global observational study summarized that the prevalence of vitamin D deficiency was 47.9% between 2000 and 2022 [1]. Vitamin D possesses a broad repertoire of functions [2], and its deficiency has been related to diverse pathologies like secondary hyperparathyroidism, bone loss [3], chronic inflammation states [4], or immunodeficiency disorders [5]. A recent study highlighted additional benefits of vitamin D supplementation in increasing the efficacy of COVID-19 vaccines (ChAdOx1 nCoV-19) by enhancing T-cell activation, proliferation, and T-cell memory responses [6].

The primary source of vitamin D in humans is UVB radiation, which stimulates the skin to produce vitamin D3 (cholecalciferol) from its precursors. Vitamin D3 then undergoes two consecutive hydroxylation steps, first occurring in the liver to form 25-hydroxyvitamin D3 (25(OH)D3, calcifediol, calcidiol) and second mainly in the kidney to generate the active 1,25-hydroxyvitamin D3 form (calcitriol) [7]. Calcitriol, as recently described for calcifediol, induces rapid non-genomic and genomic responses in the target tissues [8,9].

Currently, the concentration of serum 25(OH)D stands as the most reliable indicator for assessing the cumulative contributions from cutaneous synthesis and total intake of vitamin D metabolites [encompassing dietary sources and supplements, including animal-derived 25(OH)D3 and vegetal-derived 25(OH)D2] [10]. 25(OH)D concentrations under 20 ng/mL are considered vitamin D deficiency [11].

The present study aimed to evaluate the different strengths of new calcifediol weekly-dose formulations that would raise 25(OH)D levels of vitamin D deficient patients above optimum levels efficiently and safely.

## 2. Materials and Methods

### 2.1. Study Design

A randomized, double-blind, double-dummy, multicentre phase II/III study was conducted in two independent patient cohorts to evaluate the efficacy and safety of three weekly oral doses of calcifediol. Cohort 1 consisted of patients with 25(OH)D levels between 10 and 20 ng/mL, and Cohort 2 of patients with 25(OH)D levels of or under 10 ng/mL. In this article, we present and discuss the efficacy and safety results of weekly 75 µg and 100 µg calcifediol vs. placebo treatment in Cohort 1. The results of calcifediol dose administration of 100 µg and 125 µg in patients with severe vitamin D deficiency (Cohort 2) will be addressed in a separate manuscript.

The study was conducted in 55 sites in 7 European countries: Bulgaria (7 sites), Czech Republic (11 sites), Spain (8 sites), France (4 sites), Italy (6 sites), Serbia (6 sites) and Slovakia (13 sites) from 28 December 2020 to 25 April 2023. Independent Ethics Committees from the included countries approved the protocol before each site initiation (listed in the “Institutional Review Board Statement” section). All aspects outlined in the protocol (dosage, target population, clinical outcomes) were thoroughly reviewed and approved by the ethics committees and relevant authorities. The study was conducted in accordance with the Declaration of Helsinki. Written informed consent was received from all subjects before enrolment into the study.

### 2.2. Study Procedures

The study population consisted of males or females 18 years of age or older with serum 25(OH)D levels > 10 ng/mL and <20 ng/mL. They could not take any vitamin D or calcium supplement within the last week before screening or had it planned during the clinical study, nor could they take other drugs that could modify vitamin D levels (specified in Table S1). Females of childbearing potential had to agree to use highly effective methods of birth control and to perform pregnancy tests. Patients with severe renal impairment or diagnosed with liver or biliary failure, congestive heart failure, malabsorption, primary hyperparathyroidism, hypothyroidism, prolonged immobilization, sarcoidosis, tuberculosis, or other granulomatous diseases or hyperthyroidism were not eligible. A full list of inclusion and exclusion criteria is presented in Supplementary Table S1. A total of 398 eligible patients were randomly assigned in a 2:2:1 ratio to calcifediol 75 µg, calcifediol 100 µg or placebo, respectively. This trial was designed as a double dummy because 75 µg and 100 µg capsules differed in color. Consequently, 2 placebos or 1 verum +1 placebo per group were administered weekly. The first dose intake was on the first Sunday after randomization and continued every Sunday on a weekly basis for 52 weeks.

The primary objective of this study was to assess the efficacy of 75 µg or 100 µg of calcifediol in terms of the percentage of subjects who achieved a response defined as 25(OH)D levels ≥ 20 ng/mL and ≥30 ng/mL; at week 16 of treatment. Additional analysis of 25(OH)D levels and safety assessments were performed at weeks 4, 24, 32, and 52 for secondary objectives. A subsequent follow-up telephonic visit was performed 4 weeks after the last treatment intake. A full schedule of visits and procedures is shown in Supplementary Table S2.

Any patient with 25(OH)D levels ≤ 10 ng/mL at weeks 16, 24, or 32 was a candidate for rescue medication, receiving daily cholecalciferol 800 IU soft capsules for the rest of the study. Patients who required rescue medication continued taking the treatment assigned to their respective groups.

### 2.3. Laboratory Assessments

Pregnancy tests were performed at each visit. Blood samples were collected at baseline (screening visit) and at weeks 4, 16, 24, 32, and 52 for measurement of 25(OH)D concentration (by chemiluminescence, Elecsys Vitamin D Total II assay kit, cobas^®^, Roche Diagnostics, Rotkreuz, Switzerland), hematology, biochemistry, and other bone and mineral metabolism parameters (total serum calcium [tCa], albumin, phosphorus, PTH, and total alkaline phosphatase levels). All parameters were analyzed in a central laboratory (LKF, Laboratorium für Klinische Forschung GmbH, Schwentinental, Germany) using recognized standard methods. For the quantitative determination of total 25-hydroxyvitamin D, the Elecsys Vitamin D Total II assay employed a vitamin D binding protein (VDBP) labeled with a ruthenium complex as capture protein to bind both 25-hydroxyvitamin D3 and 25-hydroxyvitamin D2. Cross-reactivity to 24,25-dihydroxyvitamin D was blocked by a specific monoclonal antibody.

### 2.4. Statistical Analysis

The results obtained in the interim report of a previous trial with calcifediol (EudraCT no.: 2017-004028-31) were used to calculate the sample size using nQuery Advanced 8.2 software (Statistical Solutions Ltd., Cork, Ireland). A total of 57 subjects using a placebo and 114 subjects for each of the two doses of calcifediol were estimated as necessary (285 total subjects). Assuming discontinuation in approximately 20% of subjects, 355 subjects were estimated. For allocation concealment, the site staff will contact the interactive web response system via the internet to provide the subject number and 25(OH)D baseline level to get the appropriate treatment/random number from the system. A randomization list (size block: 5) was produced using the validated Statistical Analysis System (SAS) software version 9.4 for Windows (SAS^®^ Institute Inc., Cary, NC, USA).

Continuous data were summarized by the treatment group using descriptive statistics. The primary endpoint, percentage of responders, and all key secondary efficacy endpoints were analyzed with a large-sample normal approximation test of proportions (z-test). For the primary analyses, response levels of ≥30 ng/mL and ≥20 ng/mL were tested simultaneously; therefore, a multiplicity adjustment was necessary. A Bonferroni adjustment was used with a two-sided significance level of *α* = 0.0125 (0.05/4). Superiority between treatment groups was tested for all comparisons except for responders ≥ 20 ng/mL, where a non-inferiority test of 100 µg vs. 75 µg was performed. All patients receiving at least one investigational medicine and having at least one post-baseline assessment were considered for statistical analyses (Full Analysis Set, FAS). Subjects with missing values, including those not attending Visit 4 (Week 16), were considered non-responders in primary analysis but not included in secondary analyses. Additionally, all the statistical analyses were repeated in the Per Protocol Set (PPS), which was consistent with FAS subjects with no major protocol deviations that could influence the primary efficacy endpoint. Only FAS results are reported here, as the same results were obtained when comparing treatment groups in FAS or PP study populations for all evaluations performed. A subgroup analysis by BMI was performed on the primary endpoint. An MMRM (mixed model for repeated measures) model for 25(OH)D levels adjusted for treatment, visit, baseline, and including an effect for “month” was performed to directly estimate the impact that month of year has on 25(OH)D levels. For statistical significance, a *p*-value  < 0.05 was considered appropriate. SAS^®^ (version 9.4, SAS Institute Inc., Cary, NC, USA) was used for analyses within a validated and secure environment.

## 3. Results

### 3.1. Study Population

**Figure 1:**
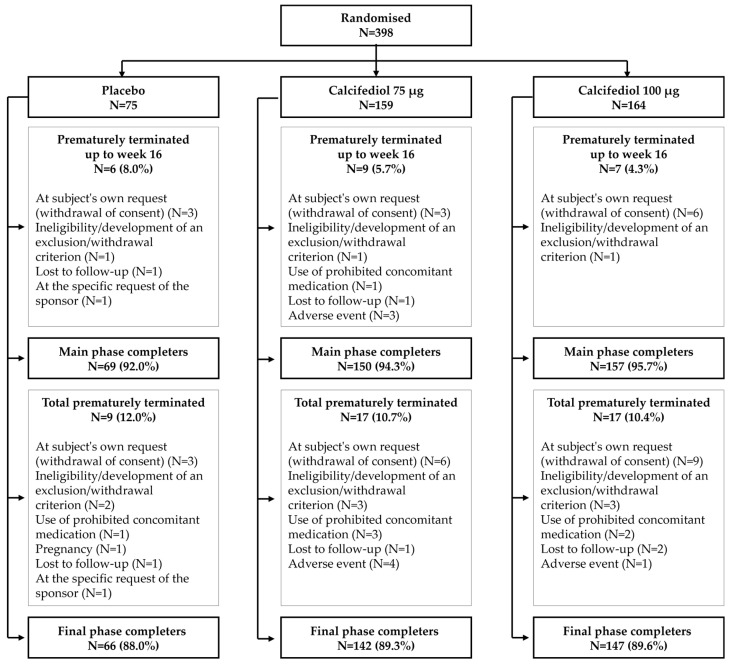
The patient flow diagram shows the number of patients randomized and conforming to each treatment group at the beginning of the study, at the completion of the main phase (week 16), and at the final phase (week 52).

From the 398 randomized patients, high compliance was achieved in the trial as a total of 376 patients (94.4%) completed 16 weeks of treatment, the timepoint of the primary assessment, and 355 subjects (89.2%) completed the 52 weeks of study. Patient disposition and prematurely terminated subjects and reasons are depicted in Figure 1. Of the total randomized subjects, 388 (97.5%) received treatment and had their 25(OH)D plasma levels assessed at least once, constituting the FAS.

Overall, the distribution of demographic and anthropometric characteristics was balanced across treatment groups (Table 1). The mean subjects’ age was 51.5 ± 16.0 years, most subjects were white (383 subjects, 98.7%), and the number of females prevailed (74.2%) with slight differences among groups (Table 1). The obese percentage of patients (BMI ≥ 30 kg/m^2^) was 23% in the group receiving placebo and comparable between both calcifediol treatment groups (30% and 33% of 75 µg and 100 µg groups, respectively). All subjects reported at least one ongoing medical condition (Table 1), with the most common metabolic disorders (dyslipidemia or diabetes type II) and vascular disorders (mainly hypertension).

Rescue medication (cholecalciferol 800 IU/d) was administered when a subject’s 25(OH)D concentration in plasma was less than 10 ng/mL at 16, 24, or 32 weeks of treatment. A total of 6 subjects (1.5%) received rescue medication during the study, including one subject from each calcifediol group and 4 subjects from the placebo group.

### 3.2. Percentage of Responders to Treatment ≥ 20 ng/mL and ≥30 ng/mL at Week 16

**Figure 2:**
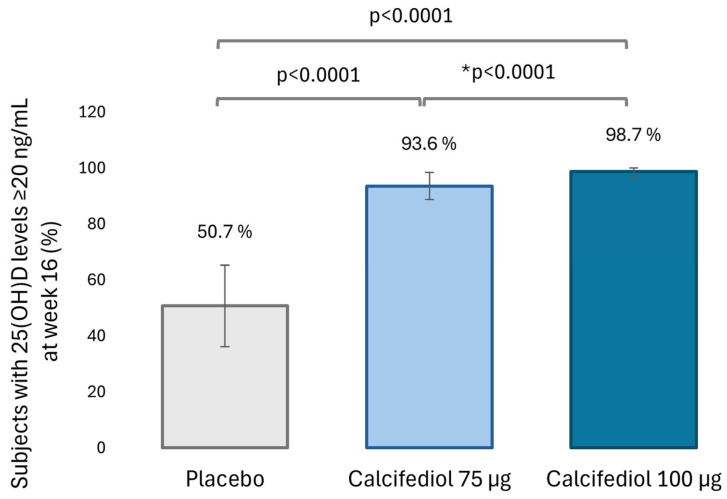
Percentage of subjects with 25(OH)D levels ≥20 ng/mL at week 16 in the placebo (grey, N = 73), 75 µg calcifediol (bright blue, N = 156) and 100 µg calcifediol (dark blue, N = 159) treatment groups. *p*-values obtained by two-sided comparisons of proportions are indicated. * *p*-value of the non-inferiority test. 98.75% confidence intervals (CI) are depicted by error bars.

**Figure 3:**
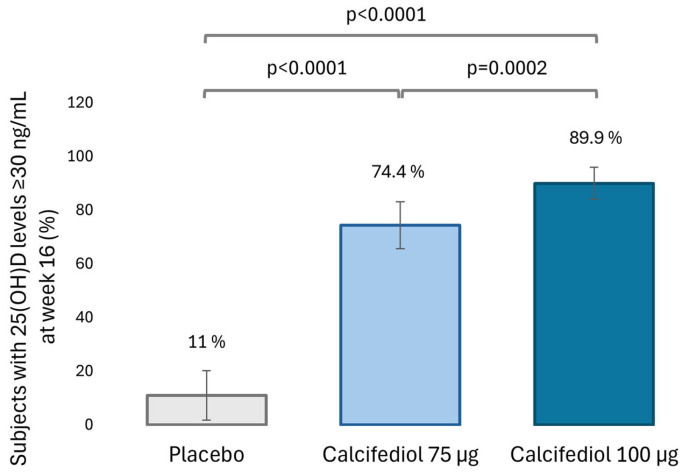
Percentage of subjects with 25(OH)D levels ≥30 ng/mL at week 16 in the placebo (gray, N = 73), 75 µg calcifediol (bright blue, N = 156) and 100 µg calcifediol (dark blue, N = 159) treatment groups. *p*-values obtained by two-sided comparisons of proportions are indicated. 98,75% confidence intervals (CI) are depicted by error bars.

Levels of 25(OH)D equal to or greater than 20 ng/mL after 16 weeks of treatment were observed in 50.7% of the placebo group (37 subjects), 93.6% of the calcifediol 75 µg group (146 subjects) and 98.7% of the calcifediol 100 µg group (157 subjects; Figure 2). The percentage of responders for 25(OH)D level ≥ 30 ng/mL at week 16 was 11.8% (8 subjects) in placebo, 74.4% (146 subjects) in calcifediol 75 µg and 89.9% (143 subjects) in calcifediol 100 µg group (Figure 3).

The superiority of both calcifediol doses compared to placebo at both response levels was demonstrated (*p* < 0.0001; 98.75% confidence) based on a two-sided test of proportions using the normal approximation. There were no significative differences between calcifediol 75 µg and 100 µg to achieve a response level of ≥ 20 ng/mL, but calcifediol 100 µg was superior to 75 µg dose in achieving a response level of ≥ 30 ng/mL (*p* = 0.0002; 98.75% confidence).

The percentage of subjects in each treatment group who achieved 25(OH)D levels of ≥20 ng/mL or ≥30 ng/mL remained similar from week 16 to the end of the study at week 52 (Figure S1).

### 3.3. Percentage of Patients Achieving a Sustained Response

**Figure 4:**
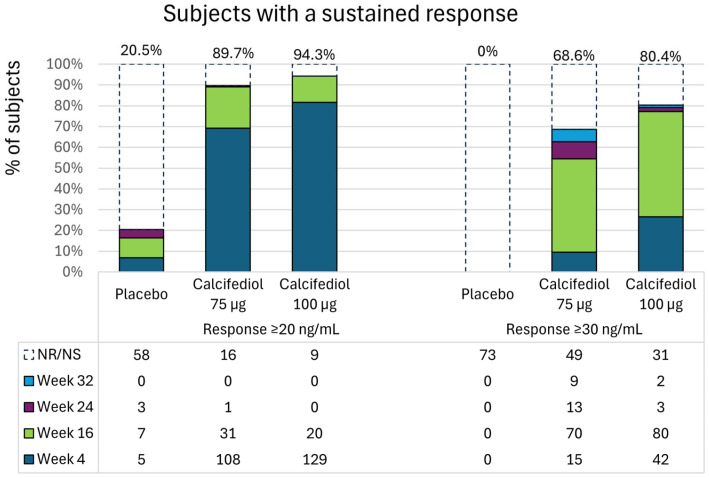
Percentage of patients starting a sustained response of ≥20 ng/mL (left) or ≥30 ng/mL (right) in the placebo, 75 µg calcifediol and 100 µg calcifediol treatment groups at week 4 (dark blue), week 16 (green), week 24 (purple) or week 32 (bright blue). The total percentage of respondents in each group is indicated in the upper part. The number of patients in each group who did not achieve a response or whose response was not sustained is denoted as Not response/Not sustained (NR/NS) in the table. In the subsequent rows, the number of subjects who achieved a sustained response is shown, grouped by the week in which this sustained response was first observed.

Sustained response to treatment was considered when a subject reaching 25(OH)D plasma levels ≥ 20 ng/mL or ≥30 ng/mL maintained levels over these thresholds in the subsequent evaluations. Most subjects in the calcifediol 75 µg group (140 subjects, 89.7%) and in the calcifediol 100 µg group (149 subjects, 94.3%) had a sustained 25(OH)D ≥ 20 ng/mL response during the study, most usually starting at week 4 (Figure 4). However, only 15 subjects (20.5%) of the placebo group had a sustained response of ≥20 ng/mL, mostly starting at week 16 (Figure 4).

For 25(OH)D ≥ 30 ng/mL response, levels were achieved and maintained over this value in 107 subjects (68.6%) in the 75 µg calcifediol group and 127 subjects (80.4%) in the 100 µg calcifediol group, mostly from week 16 in both groups (Figure 4). No subjects in the placebo group had a sustained response ≥ 30 ng/mL (Figure 4), indicating that the response obtained with a placebo was sporadic and not maintained over time.

### 3.4. Plasma 25(OH)D Levels at Different Time Points

**Figure 5:**
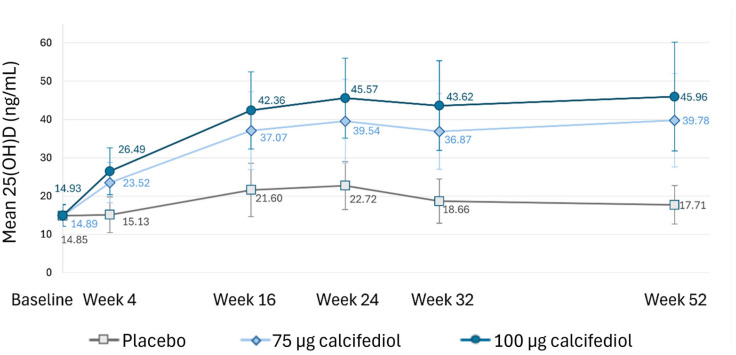
Mean plasma 25(OH)D levels (ng/mL) at each indicated timepoint for the placebo (grey), 75 µg calcifediol (bright blue), and 100 µg calcifediol (dark blue) treatment groups. Standard deviations are represented by error bars. *p* <0.0001 at every visit when comparing active treatment groups. *p* <0.0001 at every visit when comparing placebo to active treatments.

At each assessment timepoint after treatment initiation (weeks 4, 16, 24, 32 and 52), 25(OH)D concentration was calculated for placebo, 75 µg calcifediol and 100 µg calcifediol treatments to evaluate the magnitude of the response over time (Figure 5). Similar 25(OH)D mean baseline values were observed in all treatment groups: placebo 14.85 ± 2.73 ng/mL, calcifediol 75 µg 14.89 ± 2.77 ng/mL and calcifediol 100 µg 14.93 ± 2.86 ng/mL. However, values differed statistically at every timepoint after treatment initiation between placebo and calcifediol treatments (*p* < 0.0001; Figure 5) and between calcifediol formulations (*p* < 0.0001; Figure 5). In both calcifediol-supplemented groups, the mean level increased until week 24, and it remained nearly stable until the end of the study at week 52.

### 3.5. Additional Efficacy Analyses: BMI Subgroups and Monthly Modelling

As an additional exploratory analysis, the primary endpoint of the study, the percentage of subjects achieving 25(OH)D response levels ≥ 20 ng/mL and ≥30 ng/mL at week 16, was assessed regarding body mass index (BMI) subgroups. Subjects with BMI ≥ 18.5 and <25 kg/m^2^ were classified as “normal weight” (n = 126), with BMI ≥ 25 and <30 kg/m^2^ as “overweight” (n = 136), and ≥30 kg/m^2^ as “obese” (n = 116) [12]. Only 9 subjects were categorized as underweight (BMI < 18.5 kg/m^2^). Thus, no comparison analysis was performed for this category. The percentage of responders for both levels of response in each calcifediol treatment group (75 and 100 µg calcifediol) was equilibrated within BMI subgroups, and no significant differences were observed in a pairwise two-sided test of responder proportions between normal-weight, overweight and obese subgroups (Figure S2).

Sunlight exposure is the main exogenous factor affecting 25(OH)D plasma levels. Modeling of 25(OH)D level fluctuations within each treatment group (placebo, calcifediol 75 µg, and calcifediol 100 µg) was performed regarding the month of the year the assessment of 25(OH)D concentration was conducted. It reveals that the month of the year in which the 25(OH)D concentration was measured significantly affects the level observed (*p* < 0.0001), with maximum 25(OH)D levels achieved in August and September in all groups.

### 3.6. Safety

A total of 393 subjects received at least one treatment dose (safety set) and were followed up for safety evaluations until 30 days after the last treatment intake at week 52 or at an early discontinuation visit if required. A total of 148 subjects (37.7%) experienced 323 treatment-emergent adverse events (TEAEs). The overall frequency of TEAEs was comparable between placebo (38.4%), calcifediol 75 µg (33.5%), and calcifediol 100 µg (41.4%) groups (Table 2). The incidence of infections and infestations occurred in 23.3% of subjects in the placebo group, 17.1% in the calcifediol 75 µg group, and 22.8% in the calcifediol 100 µg group. Overall, subjects suffering infections and infestations, none of which were considered treatment-related, accounted for 54.7% of total subjects who experienced any TEAE. The most frequent individual TEAEs by MedDRA Preferred Term were COVID-19 (39 subjects, 9.9%), blood 25(OH)D decreased (8 subjects, 2.0%), and hypertension (7 subjects, 1.8%), mainly considered mild or moderate. A total of 16 TEAEs reported by 13 subjects (3.3%) were assessed as related to treatment, with the highest incidence of related TEAEs observed in the placebo group (9 subjects). The one TEAE possibly related to treatment in the 75 µg calcifediol group was dry mouth, while the three related TEAEs in the 100 µg group were decreased blood 25-hydrohycholecalciferol, upper abdominal pain, and chest discomfort. Seven subjects in the placebo group experienced related TEAEs of decreased blood 25-hydrohycholecalciferol, and two subjects experienced constipation. No deaths occurred in this cohort during the study. A total of 16 subjects experienced 18 serious adverse events, none of them considered treatment-related. Additionally, six subjects (1.5%) experienced TEAEs leading to discontinuation, all assessed as unrelated to the treatment.

No major mean changes from baseline within each treatment group and no relevant differences between the treatment groups were observed in vital signs, physical examinations, or hematology and biochemistry variables. Regarding the bone and mineral metabolism parameters, although no significant mean changes were observed (Table 3), slightly high levels of calcium (tCa from 10.5 to 11.9 mg/dL) [13] were reported for five subjects in the calcifediol treatment groups, 2 in the calcifediol 100 µg group and 3 in the calcifediol 75 µg group, who were discontinued from the trial according to selection criteria. The maximum level of tCa reached was 10.7 mg/dL, and in none of the subjects, it was associated with elevated 25(OH)D levels. 25(OH)D levels over the study safety cutoff of ≥80 ng/mL were reported for four subjects (1.0%) at week 52: one from the calcifediol 75 µg group (97.42 ng/mL) and three from the calcifediol 100 µg group (80.48 ng/mL; 90.32 ng/mL; 100.04 ng/mL). None of them present related adverse events. No toxic 25(OH)D levels higher than 120 ng/mL were reported.

## 4. Discussion

The present randomized, double-blind, placebo-controlled study has addressed the efficacy and safety of new weekly 75 and 100 µg calcifediol formulations in patients with baseline 25(OH)D concentrations between 10 and 20 ng/mL. Patients with vitamin D deficiency require treatment to prevent associated health risks, including secondary hyperparathyroidism, impaired respiratory and immune responses, increased bone turnover, and higher fracture risk [14], as well as to avoid further severity of vitamin D deficiency.

Active vitamin D receptors are present in various tissues, so it is unsurprising that the vitamin D endocrine system (VDES) has diverse physiological effects, each potentially requiring a specific serum 25(OH)D concentration threshold [15]. This multifunctionality, coupled with limited clinical data on the benefits of different 25(OH)D levels, has prevented consensus on an optimal threshold. The Institute of Medicine (IOM) of the United States defines sufficient levels of 25(OH)D as above 20 ng/mL [16], while some authors and scientific associations recommend levels greater than 30 ng/mL to ensure bone health, particularly in individuals with osteoporosis or those at high risk of vitamin D deficiency such as older adults [17,18,19].

In this study, the percentage of patients that reached 25(OH)D levels ≥ 20 ng/mL or ≥30 ng/mL was significantly higher after weekly administration of calcifediol 75 µg, or 100 µg than placebo. It is remarkable that most subjects restored levels above 20 ng/mL as soon as at 4 weeks of treatment and above 30 ng/mL at week 16. Although it is possible that this achievement occurred before this time, this trial lacks intermediate assessments between weeks 4 and 16. It can also be noticed that the percentage of subjects achieving levels ≥ 20 ng/mL does not differ significantly among calcifediol treatments at week 16. The reason is that this slight increase in 25(OH)D levels evaluated, from 10 to 20 ng/mL to ≥20 ng/mL, could be effectively achieved with both concentrations of calcifediol, with response rates close to 100% maximum. For this reason, a non-inferiority test was defined in the protocol as the primary endpoint for the comparison of responder rates of ≥20 ng/mL between calcifediol treatments. The effect of dose on the efficacy of calcifediol could be observed in the proportion of patients achieving a response ≥30 ng/mL since it was statistically higher in patients treated with calcifediol 100 µg than in those treated with 75 µg, and, additionally, when comparing 25(OH)D levels after each calcifediol treatment at the same time points.

The high response rate in the placebo group at week 16 for 25(OH)D levels ≥ 20 ng/mL was initially unexpected. However, upon analysis by month, this response was primarily observed during the summer (mainly August and September), coinciding with increased sunlight exposure. When the greatest percentage of placebo-treated subjects achieved values above 20 ng/mL, it is important to note that what makes an increase in 25(OH)D clinically relevant is not sporadic rise but sustained optimal plasma levels over time. In this regard, the placebo group showed lower or no sustainability in response. This suggests that fluctuations in endogenous 25(OH)D levels are common and sporadic, further supporting the need for continued vitamin D supplementation to maintain consistent optimal levels, as previously described [20].

Furthermore, treatment with the two doses of calcifediol led to an increase in 25(OH)D plasma concentrations until week 24, when a stable level was achieved and maintained over time. This steady state has been previously described for calcifediol treatments [21,22,23]. In a study by Vaes et al., adults over 65 years of age with baseline 25(OH)D levels similar to those included in this trial (10–20 ng/mL) were supplemented daily with calcifediol. They observed that plateau levels of 25(OH)D were reached with 10 µg/day (around 35 ng/mL) and 15 µg/day (around 43.2 ng/mL), which are comparable to the levels observed in this study for 75 µg/week (around 38 ng/mL) and 100 µg/week (around 44 ng/mL), respectively. Previous studies, such as those by Bischoff-Ferrari et al., who treated postmenopausal women with calcifediol at doses of 20 µg/day or 140 µg/week [24], and by Minisola et al., who evaluated three different calcifediol dosages over 3 months [25], have also reported comparable efficacy and safety between daily and weekly supplementation strategies. Additionally, a systematic literature review and meta-analysis comparing medication adherence rates between once-weekly and once-daily dosing regimens in patients with chronic disease found significantly greater adherence with weekly administration [26], positioning weekly calcifediol formulations as favourable clinical alternatives.

No significative differences in the percentage of responders ≥20 ng/mL or ≥30 ng/mL were observed within each treatment group between subjects categorized by BMI. These results align with a study by Charoenngam et al., which found no significant difference in systemic 25(OH)D bioavailability (AUCs) after a single 900 µg dose of calcifediol between higher and lower BMI groups [27]. Similarly, no differences were observed in 25(OH)D levels between obese and non-obese postmenopausal women following 0.266 mg/month calcifediol treatment for 12 months [22]. The literature largely supports the high efficacy of calcifediol across all BMI subgroups.

The establishment of a consensus on the maximum safe serum 25(OH)D level remains controversial. Some data suggest that levels below 100 ng/mL are not associated with toxicity [28], while other studies indicate that toxicity is unlikely unless 25(OH)D concentrations exceed 120 ng/mL or 150 ng/mL [29,30,31]. For our study, we set a more conservative safety cut-off at 80 ng/mL. After 52 weeks of calcifediol treatment, four patients exceeded 80 ng/mL (maximum level = 100.04 ng/mL), but none reached toxic levels (>120 ng/mL). No treatment-related adverse events were reported in patients with 25(OH)D levels above 80 ng/mL.

The primary adverse effect of hypervitaminosis D is hypercalcemia, defined as total calcium levels (tCa) of 10.5 mg/dL or higher, according to standard laboratory references [16]. Hypercalcemia can result from excessive consumption of calcium or vitamin D, though it is more commonly linked to conditions like primary hyperparathyroidism [32]. In this study, five subjects in the calcifediol groups were withdrawn due to elevated tCa values (maximum tCa = 11.7 mg/dL). None reached serum calcium levels above 12 mg/dL, the threshold where kidney calcium reabsorption may be compromised, leading to hypercalciuria [33]. No hypercalciuria-related adverse events were reported, and none of the patients with elevated tCa had raised 25(OH)D levels. The long-term safety of calcifediol has been previously documented [22,34], including over a two-year period [35]. Since the incidence of treatment-emergent adverse events (TEAEs) was comparable between placebo and calcifediol weekly treatments, with no serious treatment-related adverse events and no relevant changes from baseline in vital signs, physical exams, haematology or lab results, weekly doses of 75 µg or 100 µg calcifediol are considered safe for long-term use.

A limitation of this study is the lack of comparison between weekly calcifediol and an active comparator, such as monthly calcifediol or a non-hydroxylated form of vitamin D_3_ cholecalciferol. Previous studies have shown that cholecalciferol increases 25(OH)D plasma levels more slowly and is less potent, often requiring higher doses for effective treatment [21,34,36]. Additionally, oral calcifediol has a higher intestinal absorption rate, and this may have important advantages in cases of reduced intestinal absorption due to various gastrointestinal conditions. Another limitation is the lack of calcium correction for albumin levels, which some consider a more accurate estimation [37], although others argue that uncorrected total calcium values better reflect biologically active calcium [38]. On the other hand, the study has several strengths, including a 52-week observational period, a large sample size of 398 patients with homogenous demographics, and high completion rates of 94.4% at 16 weeks and 89.2% at one year. Treatment compliance was also notably high, exceeding 90% at both 16 weeks and 52 weeks, which adds robustness to the trial.

## 5. Conclusions

In conclusion, the results of this study demonstrate that the new weekly formulations of calcifediol, at doses of 75 µg and 100 µg, are superior to placebo in achieving optimal 25(OH)D levels in patients with vitamin D deficiency (defined as 25(OH)D levels between 10 and 20 ng/mL). Both long-term weekly calcifediol treatments resulted in a stable and sustained response within optimal levels throughout the 52-week study period, with a favourable safety profile. These findings support the clinical use of the 75 µg and 100 µg weekly formulations of calcifediol as effective initiation and long-term maintenance therapies for patients with vitamin D deficiency, thereby helping to prevent complications related to hypovitaminosis D. Furthermore, weekly calcifediol treatments may enhance patient acceptance and adherence to therapy.

## Figures and Tables

**Table 1:** Mean baseline demographic and clinical data. BMI, body mass index; µg, micrograms; n, number of subjects; SD, standard deviation.

Variable	Placebo (n = 73)	Calcifediol 75 µg (n = 156)	Calcifediol 100 µg (n = 159)	Total (n = 388)
Age, years, mean (SD)	50.1 (15.4)	52.4 (15.8)	51.2 (16.4)	51.5 (16.0)
Sex, female, n (%)	51 (69.9)	123 (78.8)	114 (71.7)	288 (74.2)
BMI, mean (SD) kg/m^2^	27.2 (6.2)	27.6 (5.1)	28.1 (6.3)	27.7 (5.8)
BMI, n (%):				
<18.5 kg/m^2^	0	2 (1.3)	7 (4.4)	9 (2.3)
≥18.5, <25 kg/m^2^	32 (43.8)	48 (30.8)	46 (28.9)	126 (32.5)
≥25, <30 kg/m^2^	23 (31.5)	60 (38.5)	53 (33.3)	136 (35.1)
≥30 kg/m^2^	17 (23.3)	46 (29.5)	53 (33.3)	116 (29.9)
Main comorbidities:				
Dyslipidaemia, n (%)	13 (17.8)	24 (15.4)	27 (17.0)	64 (16.5)
Type 2 diabetes, n (%)	6 (8.2)	16 (10.3)	20 (12.6)	42 (10.8)
Hypertension, n (%)	19 (26.0)	56 (35.9)	56 (35.2)	131 (33.8)

**Table 2:** Safety summary. Number (n) and percentage (%) of subjects in the safety population of each treatment group (N) who experienced the indicated number (E) of treatment-emergent adverse events (TEAE).

	Placebo (N = 73)		Calcifediol 75 µg (N = 158)		Calcifediol 100 µg (N = 162)		Total (N = 393)	
	n (%)	E	n (%)	E	n (%)	E	n (%)	E
TEAE	28 (38.4)	57	53 (33.5)	136	67 (41.4)	130	148 (37.7)	323
Non-serious TEAE	27 (37.0)	49	52 (32.9)	129	66 (40.7)	127	145 (36.9)	305
Serious TEAE	6 (8.2)	8	7 (4.4)	7	3 (1.9)	3	16 (4.1)	18
Related TEAE	9 (12.3)	10	1 (0.6)	1	3 (1.9)	5	13 (3.3)	16
Related serious TEAE	0	0	0	0	0	0	0	0
Severe TEAE	6 (8.2)	7	8 (5.1)	9	2 (1.2)	2	16 (4.1)	18
TEAE leading to discontinuation	1 (1.4)	1	4 (2.5)	7	1 (0.6)	1	6 (1.5)	9

**Table 3:** Summary of mean changes from baseline in bone and mineral metabolism parameters at week 16 and week 52. The number of subjects in the Safety Set (N), number of subjects with data available (n), and mean and standard deviation (SD) are indicated.

Parameter Visit	Placebo (N = 73)		Calcifediol 75 µg (N = 158)		Calcifediol 100 µg (N = 162)	
	n	Mean (SD)	n	Mean (SD)	n	Mean (SD)
Alkaline phosphatase (U/L)						
Week 16	69	−1.3 (14.60)	151	−3.4 (10.75)	157	−2.1 (13.51)
Week 52	65	0.3 (12.88)	142	−0.8 (12.21)	146	−0.9 (13.84)
Total serum calcium (mg/dL)						
Week 16	69	0.01 (0.301)	151	0.01 (0.318)	157	0.03 (0.0343)
Week 52	65	−0.01 (0.268)	142	0.02 (0.337)	146	0.03 (0.315)
Phosphorous (nmol/L)						
Week 16	69	0.038 (0.155)	151	0.031 (0.162)	157	0.060 (0.182)
Week 52	65	0.055 (0.151)	142	0.019 (0.176)	146	0.050 (0.174)
Parathyroid hormone						
Week 16	66	−6.5 (13.98)	140	−9.7 (16.21)	150	−10.4 (14.93)
Week 52	61	−1.4 (17.85)	137	−5.7 (13.52)	137	−8.4 (14.18)

## Data Availability

In alignment with ethical standards and best practices, we commit to sharing deidentified data from this trial. Data access will be provided to qualified researchers affiliated with academic or research institutions. Interested researchers must submit a research proposal outlining objectives and methodology to clinical_rd@faes.es for sponsor assessment. Data requestors approved by the sponsor will need to sign a data access agreement. Details on the specific secure and controlled platform to access the data will be provided in the agreement. Once signed, the data of this study will be available. Data access requests will be possible beginning 3 months and ending 5 years following article publication of primary results in a peer-reviewed journal.

## Supplementary Materials

The following supporting information can be downloaded at: https://www.mdpi.com/article/10.3390/nu16223796/s1, Table S1: Inclusion and exclusion criteria; Table S2: Schedule of events and assessments by visit; Figure S1: Percentage of subjects with 25(OH)D levels; Figure S2: Percentage of responders by BMI subgroup.
